# Bioengineered 3D Human Kidney Tissue, a Platform for the Determination of Nephrotoxicity

**DOI:** 10.1371/journal.pone.0059219

**Published:** 2013-03-14

**Authors:** Teresa M. DesRochers, Laura Suter, Adrian Roth, David L. Kaplan

**Affiliations:** 1 Department of Biomedical Engineering, Tufts University, Medford, Massachusetts, United States of America; 2 F. Hoffmann La Roche, Ltd. Non-Clinical Safety, Basel, Switzerland; Université de Technologie de Compiègne, France

## Abstract

The staggering cost of bringing a drug to market coupled with the extremely high failure rate of prospective compounds in early phase clinical trials due to unexpected human toxicity makes it imperative that more relevant human models be developed to better predict drug toxicity. Drug–induced nephrotoxicity remains especially difficult to predict in both pre-clinical and clinical settings and is often undetected until patient hospitalization. Current pre-clinical methods of determining renal toxicity include 2D cell cultures and animal models, both of which are incapable of fully recapitulating the *in vivo* human response to drugs, contributing to the high failure rate upon clinical trials. We have bioengineered a 3D kidney tissue model using immortalized human renal cortical epithelial cells with kidney functions similar to that found *in vivo*. These 3D tissues were compared to 2D cells in terms of both acute (3 days) and chronic (2 weeks) toxicity induced by Cisplatin, Gentamicin, and Doxorubicin using both traditional LDH secretion and the pre-clinical biomarkers Kim-1 and NGAL as assessments of toxicity. The 3D tissues were more sensitive to drug-induced toxicity and, unlike the 2D cells, were capable of being used to monitor chronic toxicity due to repeat dosing. The inclusion of this tissue model in drug testing prior to the initiation of phase I clinical trials would allow for better prediction of the nephrotoxic effects of new drugs.

## Introduction

It is estimated that it takes 9 years and costs between 0.8 and 1.7 billion dollars to bring a drug through clinical trials [1], [2]. Compounded upon the cost and time, only about 8% of drugs entering Phase I clinical trial testing make it to market [2]. This low rate is due to numerous factors including the lack of well-established testing methods capable of accurately predicting clinical usefulness and drug toxicity during pre-clinical development. The FDA's Critical Path Initiative calls for the development of better tools to improve the success of drug candidates that proceed from pre-clinical testing to clinical trials [2]. Included in this process is the development of human tissue models better capable of predicting human *in vivo* responses, including organ toxicity. The kidney is a major site of drug–induced toxicity as it receives about 25% of cardiac output and is a site of significant excretion. Only about 7% of drug candidates fail due to nephrotoxicity in pre-clinical testing with 2D cell cultures and animal models [3], while it is estimated that 30–50% of all cases of severe acute renal failure in patients are due to drug–induced nephrotoxicity [3], [4]. Thus, current pre-clinical testing methods are not rigorous enough to predict the human response to most drugs.

Currently, drug–induced nephrotoxicity is tested in both monolayer (2D) cell cultures [5], [6] and animal models [7], [8]. 2D cell culture models offer the advantage of being simple and low in cost. However, 2D cell cultures are incapable of recapitulating the complexity of the *in vivo* environment [9], [10] and have been shown to often require higher doses over longer time periods to induce a toxic response compared to *in vivo* toxicity responses in patients [11], [12]. Animal models provide the benefit of a complex system that is lost with 2D cell cultures. However, animal biology differs in many respects from human biology due to differences in physiology and environment, thus making them capable of predicting human responses only to a certain extent [13]. By default animal trials, in particular rodent studies, are highly ordered with the same genetic backgrounds, ages, environmental factors, and disease states examined in large numbers with many testing methods. This is not the case with human drug testing as genetic backgrounds, ages, and environmental factors cannot be as rigorously controlled, especially in early human trials. Importantly, in the case of drug–induced nephrotoxicity, the people most susceptible are those with underlying kidney conditions or currently taking other nephrotoxic drugs. These people are normally not included in clinical trials and so their response is not known until they are actually treated with an approved drug. Animal models are also expensive and pose an ethical issue that is currently being addressed by the principles of 3Rs, replace, reduce, and refine. Bioengineered tissues with human cells are expected to better reflect the situation in patients, as they will allow for acute toxicity testing but also for longer exposures. This may reveal cumulative tissue injury with repeated administration of subtoxic concentrations; a situation more relevant to the clinical practice. Moreover, 3D tissues undergoing compound exposure can be used to detect biomarkers indicative of nephrotoxicity *in vivo*, thus enabling the translation from the *in vitro* to the *in vivo* situation. Bioengineered 3D tissues have been used for toxicity testing for both human liver [14], [15], [16], [17] and skin [18], [19], [20], but a 3D system for human nephrotoxicity testing does not currently exist. Here we describe a bioengineered 3D human renal tissue system and its assessment as a predictor of human nephrotoxicity. The bioengineered tissue is functionally and morphologically similar to human kidney tissue *in vivo*. Treatment of the tissue with known nephrotoxicants showed that the 3D tissue was more sensitive to lower drug concentration than the same cells grown in 2D. Additionally, long term studies revealed the utility of the 3D model for chronic toxicity studies over the 2D system.

## Materials and Methods

### Cell culture

The NKi-2 cells used in all studies were derived by serial passaging in low serum media of hTERT immortalized human renal cortical cells, a generous gift from Dr. Jing Zhou. These human renal cortical epithelial cells were isolated from patients following written consent and approval from Brigham and Women's Hospital (IRB #2009-P-000742/7) and immortalized by infection with hTERT. Cells were grown in high glucose DMEM/F12 (Invitrogen) containing 2% FBS (Invitrogen), 20 ng/mL hEGF (Invitrogen), 72 ng/mL T3 (Sigma), 1% ITS (Invitrogen), 100 ng/mL hydrocortisone (Sigma), and 1% penicillin/streptomycin (Invitrogen). For MTT assay, NKi-2 cells were seeded at a density of 5000 cells/well of a 96-well cell culture plate 24 hours prior to assay initiation. Cisplatin (Sigma) was dosed in a 10-fold range from 0.01 µM to 100 µM, gentamicin (Invitrogen) was dosed at 0.2, 1.1, 2.2, 10.8, and 21.6 mM, and doxorubicin (Sigma) was dosed at 0.001 µM and a 10-fold range from 0.02 µM to 20 µM for 48 hours. MTT reagent (Invitrogen) was added to each well for 5hrs at 37 °C. Cells were lysed with DMSO for 15 minutes at 37 °C and absorbance was read at 570 nm.

### Immunocytochemistry and immunohistochemistry

For immunocytochemistry, cultures were grown on cover slides and fixed with 4% paraformaldehyde in PBS for 5 minutes. Cells were immunostained with rabbit anti-E-cadherin (Abcam), mouse anti-Cytokeratin 8/18/19 (Abcam), mouse anti-GGT1 (Abcam), mouse anti-SLC22A6 (OAT1) (Abcam), or rabbit anti-SLC22A11 (OAT4) (Abcam) for 1 hour followed by Alexa fluor 594-conjugated goat anti-rabbit secondary antibody or Alexa fluor 488-conjugated goat anti-mouse secondary antibody (Invitrogen) for 30 minutes at room temperature. Cells were counterstained with DAPI in Vectashield mounting medium (Vector Labs). For Immunohistochemistry, tissues were fixed with 10% buffered formalin for 48 hours and embedded in paraffin. Eight micron sections underwent antigen retrieval in citrate buffer and were stained in the same manner as for immunocytochemistry. Tissue sections were counterstained with DAPI in Vectashield mounting medium (Vector Labs). For tissue morphology, tissues were fixed with 10% buffered formalin and either embedded in paraffin for H&E staining or stained with carmine (Sigma) for whole mounts. H&E staining was performed on 8 µm tissue sections. Immunofluorescent and H&E images were captured with Leica Application Suite v4, using a Leica DMIL microscope equipped with a Leica DFC340FX camera for fluorescent images and a Leica DFC295FX camera for brightfield images.

### 3D tissue constructs

3D tissue constructs were formed in 12-well transwell dishes with 0.4 µm porous polycarbonate membranes (Corning) utilizing both Matrigel (BD Biosciences) and rat tail collagen I (BD Biosciences). Briefly, each transwell insert was coated with an acellular layer of a 50:50 mix of Matrigel and rat tail collagen I at a final concentration of 1 mg/mL. After polymerization, 100,000 NKi-2 cells were mixed with a 50:50 mix of Matrigel and rat tail collagen I at a final concentration of 1 mg/mL and added onto each coated transwell insert. The tissues were allowed to polymerize at 37 °C for 1 hour after which NKi-2 cell culture media was added to both the bottom well of the transwell and inside the insert. Tissues were grown at 37 °C, 5% CO_2_ with media changes every 2 days.

### Functional assays

**Hydrolase activity** was determined as follows, for γ-glutamyl transpeptidase (GGT1) activity, NKi-2 cells were plated at 1,280 cells/well of a 96-well plate and grown for 7 days. On the day of assay initiation, the cells were washed with PBS and incubated in 200 µL of substrate reagent (2.5 mM L-γ-glutamyl-p-nitroanilide, Tris-HCl pH 8.6, 150 mM NaCl, 50 mM glycylglycine) for 1 hour at room temperature. Absorbance was read at 405 nm on a SpectraMax M2 plate reader (Molecular Devices) and human foreskin fibroblasts (Lonza) were used as controls. For leucine aminopeptidase (LAP) activity, NKi2 cells were treated the same as for GGT1 activity, but the substrate reagent consisted of 3 mM L-leucine-p-nitroanilide in PBS.

**Adenylate cyclase** activity was determined as follows, NKi-2 cells were seeded at 7,600 cells/well of a 24 well plate and grown to confluency and 3D tissues were made as described and grown for 2 weeks. On the day of assay initiation, media was removed and cells and tissues were washed 2 times with HBSS (Invitrogen). Cells and tissues were incubated with 0.2 µM parathyroid hormone (Sigma), 1 µM antidiuretic hormone (Sigma) or 25 µM forskolin for 30 minutes at 37 °C, 5% CO_2_. Cells and tissues were lysed and cyclic AMP concentration was quantified with a competitive binding assay from R&D systems following the manufacturer's instructions.

**Glucose uptake** in both the NKi-2 cells and 3D tissues was determined as follows; NKi-2 cells were plated at 100,000 cells/well of a 6-well plate and grown to confluency. 3D tissues were prepared as described and grown for 2 weeks. On the day of assay initiation, cells and tissues were washed 2 times with incubation buffer (137 mM NaCl, 5.4 mM KCl, 1.4 mM CaCl_2_, 0.6 mM MgSO_4_, 10 mM HEPES, pH7.4). Incubation buffer without NaCl was used for the no sodium condition. Treatment groups included, incubation buffer with 1 mM 2-deoxyglucose (Sigma), incubation buffer without NaCl with 1 mM 2-deoxyglucose, and incubation buffer with 1 mM 2-deoxyglucose, 1 mM phlorizidin (Sigma) as a control. Cells and tissues were incubated in their treatment for 1 hour at 37 °C, 5% CO_2_. After incubation, all samples were washed 4 times. Treatment groups with sodium were washed in 137 mM NaCl, 20 mM HEPES, pH 7.4 and treatment groups without sodium were washed in 20 mM HEPES, pH 7.4. Cells and tissues were lysed with 0.5% triton X-100 at 80 °C for 15 minutes and centrifuged at 15,000 g for 20 minutes at 4 °C. Glucose uptake was assayed with a kit from Cosmobio (#CSR-OKP-PMG-K01TE) following the manufacturer's instructions.

### RT-PCR

For RT-PCR, RNA was extracted from both NKi-2 cells grown on 10 cm^2^ cell culture dishes and 2 week 3D tissues using RNeasy kit from Qiagen. Transcripts were amplified from 50 ng of RNA using Superscript one-step RT-PCR system from Invitrogen with an annealing temperature of 57 °C and 35 cycles. The following primers were used: MDR1 (forward) CCTAGGAGTACTCACTTCAGGA, (reverse) AAGATCCATTCCGACCTCGC, MRP2 (forward) ACGCAGTCCAGGAATCATGC, (reverse) AAAACCAGGAGCCATGTGCC, MRP4 (forward) GTGGCCGTGATTCCTTGGAT, (reverse) GGCATCCAGAGTTTTTGCCAG, MRP5 (forward) CTGAAGCCCATCCGGACTAC (reverse) CACCATGAAGGCTGGTCCAC, and megalin (forward) TGGATGTGAAAGCGGTCCTC, (reverse) ACTCAACACAGGTACGGCTG. Products were run on a 2% agarose gel containing ethidium bromide and imaged on a G:BOX gel imager from Syngene.

### DNA content and secretion of LDH, Kim-1, and NGAL

In order to determine DNA content overtime, tissues were made as described. Tissues were removed from culture at 14, 17, 21, 24, and 28 days, lysed in 5 mM MgCl_2_, 0.2% triton X-100, centrifuged at 12,000 rpm for 10 minutes at 4 °C, and DNA was quantified using Quant-iT picogreen dsDNA assay kit from Invitrogen following the manufacturer's instructions. LDH secretion was assayed in cell culture supernatant from both NKi-2 cells and 3D tissues using LDH cytotoxicity assay from Clontech and following the manufacturer's instructions. Kim-1 and NGAL secretion was assayed in the cell culture supernatant from both the NKi-2 cells and 3D tissues using ELISA assays from R&D systems and following the manufacturer's instructions. All measurements were performed with Softmax Pro 5.4 software on a SpectraMax M2 plate reader (Molecular Devices).

### Statistics and LD_50_ calculations

Statistical analyses were performed using GraphPad Prism 5 software. Two-way analysis of variance was used with Bonferroni tests to examine differences between treatment groups. Statistical significance was set at p<0.05 and all values are expressed as mean +/− standard error of the mean. LD_50_, the dosage at which 50% toxicity was achieved, was calculated using LDH data on day 3 of treatment for 2D cells and day 7 of treatment for 3D tissues as these were the days of maximal toxicity at the highest drug concentrations.

## Results

### Characterization of Phenotype and Function of Human Renal Epithelial Cells

**Figure 1:**
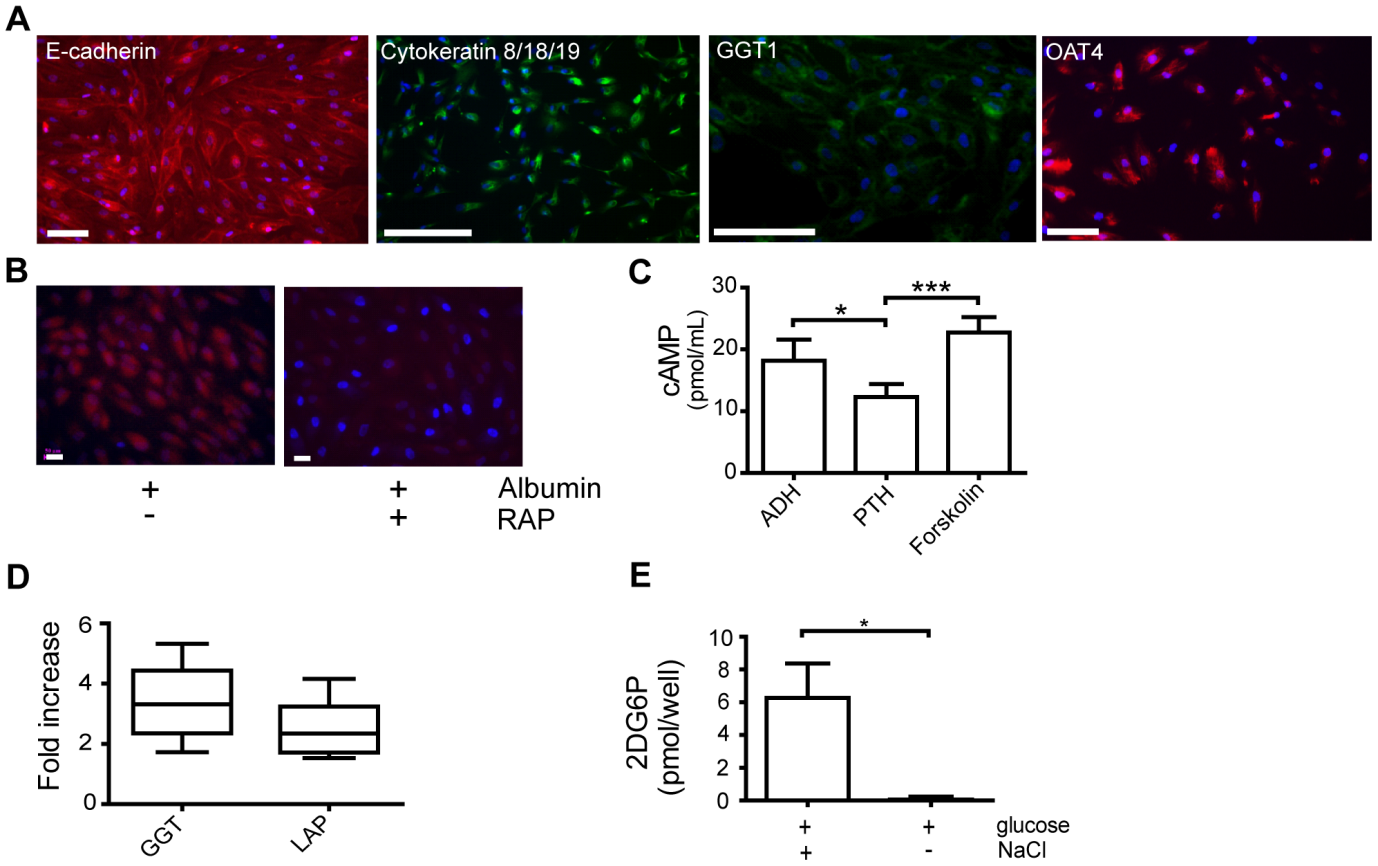
Human renal proximal tubule cells maintain epithelial markers and function with immortalization and 2D cell culture. (A) Expression of epithelial cell markers, E-cadherin (red) and cytokeratin 8/18/19 (green), the proximal tubule cell marker GGT1 (green), and the organic anion transporter OAT4 (red) in NKi-2 cells. Nuclei are stained with dapi (blue). Scale bars  =  100 µm. (**B**) Adenylate cyclase activity in NKi-2 cells upon treatment with ADH or PTH. Treatment with forskolin acted as a control for production of cAMP. *p<0.05, ***p<0.0001, n  =  8. (**C**) GGT and LAP hydrolase activity in NKi-2 cells. Fold increase is calculated against human fibroblast activity. n  =  48. (**D**) Na^+^ dependent glucose uptake in NKi-2 cells. Cells were exposed to 2-DG with or without Na^+^ in the form of NaCl. *p<0.05, n  =  5.

Human renal cortical epithelial cells immortalized through the incorporation of hTERT were used for the development of the 3D human kidney tissue system. These cells were selected for an epithelial cell population via growth over 10 passages in reduced serum conditions. The resulting cell population (NKi-2) was characterized for an epithelial and proximal tubule phenotype as well as organic anion transporter expression by staining for e-cadherin, cytokeratin 8/18/19, and γ-glutamyl transpeptidase (GGT1), organic anion transporter 1 (OAT1) and OAT4 (**Fig. 1A**). The NKi-2 cells failed to stain positive for OAT1 in 2D culture (data not shown), but were positive for all other examined markers. To examine the ability of the NKi-2 cells to function as renal proximal tubule cells, they were subjected to multiple functional assays including adenylate cyclase activity, hydrolase activity, and glucose uptake [21], [22]. Renal proximal tubule cells respond to parathyroid hormone (PTH) by up-regulating adenylate cyclase activity thus producing cyclic AMP. They do not respond to antidiuretic hormone (ADH) in the same manner [23]. To test for the NKi-2 cell's ability to properly respond to PTH, they were incubated with PTH, ADH, or forskolin, a control for adenylate cyclase activation, for 30 minutes and assayed for cyclic AMP expression (**Fig. 1B**). In 2D culture, the NKi-2 cells did produce cyclic AMP in response to PTH as would be found *in vivo*. However, they produced a significantly (p<0.05) larger amount in response to ADH which is not representative of normal renal proximal tubule cell (RPTC) function. We also examined hydrolase activity by examining the activity of both GGT1 and leucine aminopeptidase (LAP) on the cell surface (**Fig. 1C**) as this activity plays a role in the metabolism of drugs such as cisplatin [24]. A 4 fold-increase in GGT1 activity in the NKi-2 cells compared to a normal human fibroblast control and a 2-fold increase in the activity of LAP were found, indicating active hydrolases on the surface of the NKi-2 cells. Finally, RPTC uptake glucose in the presence of sodium [25]. To test the ability of the NKi-2 cells to uptake glucose, the cells were incubated with 2-deoxy-glucose (2-DG6P) with or without NaCl and assayed for the amount of glucose absorbed by the cells (**Fig. 1D**). The NKi-2 cells functioned as seen *in vivo* by only absorbing glucose in the presence of NaCl. These results indicated that the human RPTC line, NKi-2, was capable of mimicking many proximal tubule cell functions.

### Establishment and Characterization of Bioengineered 3D Human Renal Tissue Constructs

**Figure 2:**
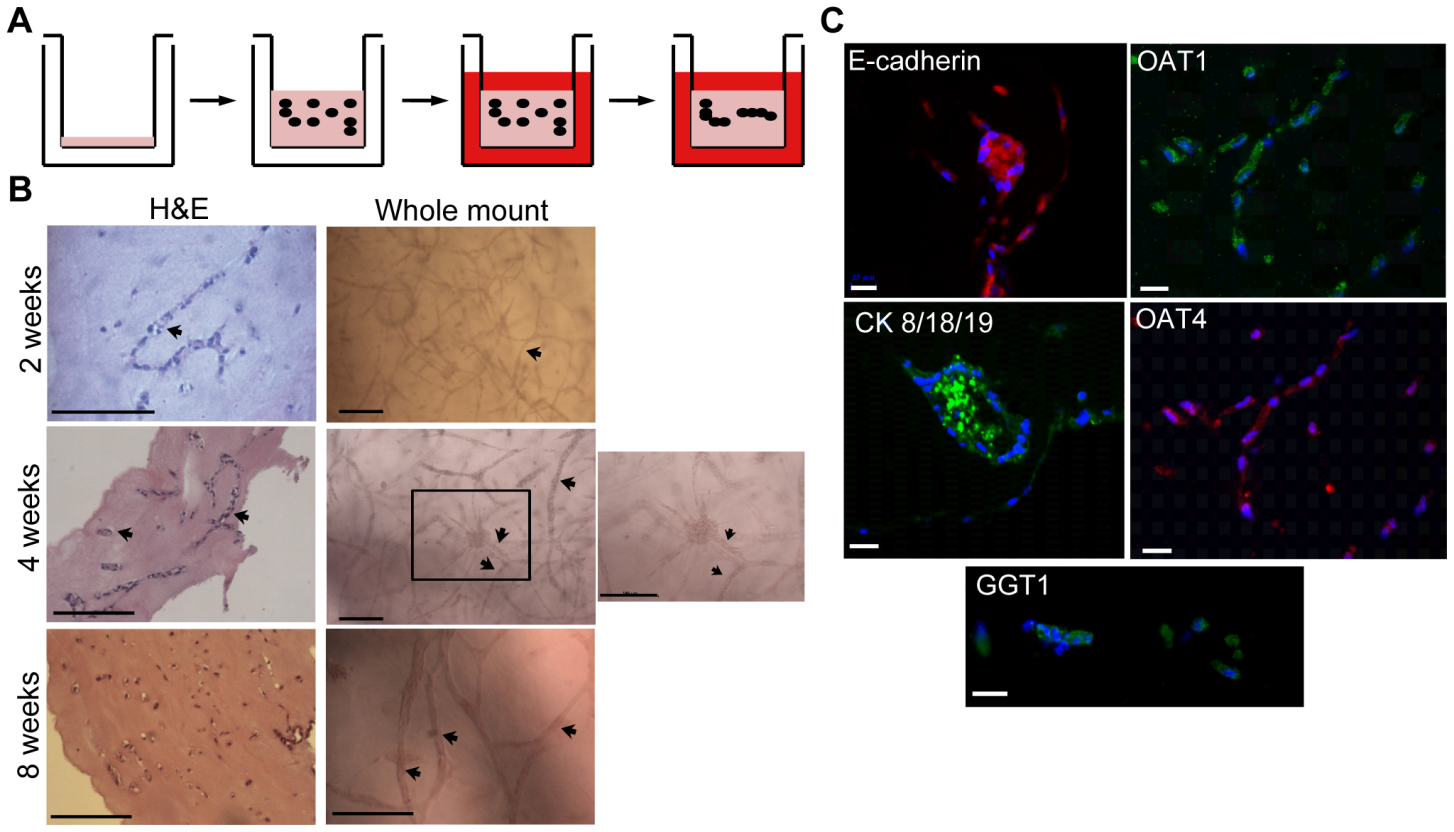
NKi-2 cells formed tubular structures when grown in 3D tissues. (A) Schematic of 3D tissue formation. An acellular layer of 1:1 Matrigel:rat tail collagen I (1 mg/mL) is layered onto a tran-well membrane. Following polymerization, a layer of the same ECM mixture containing NKi-2 cells is added. The tissues are maintained in growth media and after approximately 2 weeks, the cells organize into branching structures. (B) Morphology of NKi-2 cells after extended growth in 3D tissues. Tissue sections, 10 µm, were stained with H&E and whole tissue sections were stained with carmine for whole mounts. Arrows indicate areas of branching tubular-like structures. Scale bars  =  100 µm. (**C**) Expression of epithelial cell markers, e-cadherin (red) and cytokeratin 8/18/19 (green), kidney proximal tubule cell marker GGT1 (green) and organic anion transporters OAT1 (green) and OAT4 (red) within the 3D tissues after 4 weeks of growth. Scale bars  =  25 µm.

**Figure 3:**
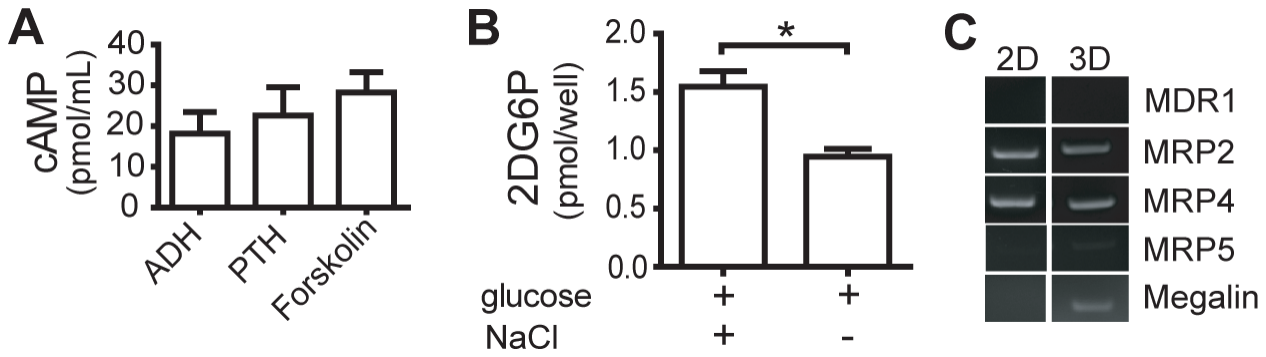
NKi-2 cells maintain kidney function when grown in 3D tissues. (A) Adenylate cyclase activity in NKi-2 cells within the bioengineered 3D tissue upon treatment with ADH or PTH. Treatment with forskolin acted as a control for production of cAMP. n  =  4. (**B**) Na^+^ dependent glucose uptake in NKi-2 cells within the bioengineered 3D tissues. Tissues were exposed to 2-DG with or without Na^+^ in the form of NaCl. *p<0.05, n  =  5. (C) RT-PCR of drug tranporters found in kidney epithelial cells using RNA from both NKi-2 cells and 4 week 3D tissues. (**C**) RT-PCR of different transport proteins found in human kidney cells in both NKi-2 cells (2D) and 4 week old 3D tissues.

Once it was established that the NKi-2 cell line was epithelial and functional, the cells were incorporated into a complex extracellular matrix (ECM) to initiate the formation of a 3D tissue. An acellular layer of 1:1 Matrigel:rat tail collagen I (1 mg/mL) was layered on the bottom of a 0.4 µm transwell and allowed to polymerize. The NKi-2 cells were mixed into an additional 1:1 solution of Matrigel:rat tail collagen I (1 mg/mL) which was layered on top of the acellular layer and allowed to polymerize to form a 3D tissue. These tissues were maintained in transwell cell culture dishes to allow media access to both the bottom and top of the tissues (**Fig. 2A**). Tissue structure, based on the formation of tubules within the ECM, was examined at different time points by both H&E staining of tissue sections and carmine staining of whole tissues (whole mount). The 3D interconnected tubular structure was established by 2 weeks and maintained for up to 8 weeks without loss of structure or overgrowth of the system, based on both H&E staining that revealed tubule structure formation and whole mount staining that showed both the prevalence of the tubules and their interconnection (**Fig. 2B**). These tissues maintained the expression of epithelial (E-cadherin and cytokeratin 8/18/19) and proximal tubule (GGT1) cell markers and also expressed OAT1 and OAT4 (**Fig. 2C**). The 3D tissues were analyzed for function by examining glucose uptake and adenylate cyclase activity. For the functional assays, the 3D tissues were formed and allowed to grow for 2–3 weeks prior to testing to ensure proper structural development. These tissues were capable of normal glucose uptake in the presence of NaCl similar to 2D culture (**Fig. 3B**). IAdenylate cyclase activity however was changed between 2D and 3D culture. Unlike the 2D cultures (**Fig. 1C**), in the 3D tissues there was no significant difference in cAMP expression between ADH, PTH, or Forskolin (**Fig. 3A**). This was due to an increase in the amount of cAMP produced in response to PTH within the 3D tissues. While this is still not fully representative of normal *in vivo* behavior, this response may indicate a more *in vivo*-like phenotype when the cells are grown in a more complex 3D environment. Finally, since the ability to transport compounds across the cell membrane is vital for a nephrotoxic response, we also looked at the expression of particular transport proteins known to function in the human kidney (**Fig. 3C**) [26]. The NKi-2 cells expressed transcripts for MRP2 and 4 in both 2D cell culture and 3D tissues, while MDR1 was not expressed in either case. Interestingly, both megalin and MRP5 were expressed in 3D tissues, but the transcripts were not detected when the same cells were in 2D monolayer culture. MRP2 and MDR1 have been shown to be involved in doxorubicin uptake [27] while megalin is involved in gentamicin uptake [28]. The difference in transporter transcript expression between 2D and 3D may relate to differences in toxicity between the different culture conditions and may indicate a more *in vivo*-like phenotype when the cells are grown in 3D as compared to 2D culture. Taken together, these results show growth of a functional bioengineered 3D human kidney tissue that is capable of both maintaining and re-expressing drug transporters.

### Development of Toxicity Assays for Use with Bioengineered 3D Human Renal Tissue Constructs

**Figure 4:**
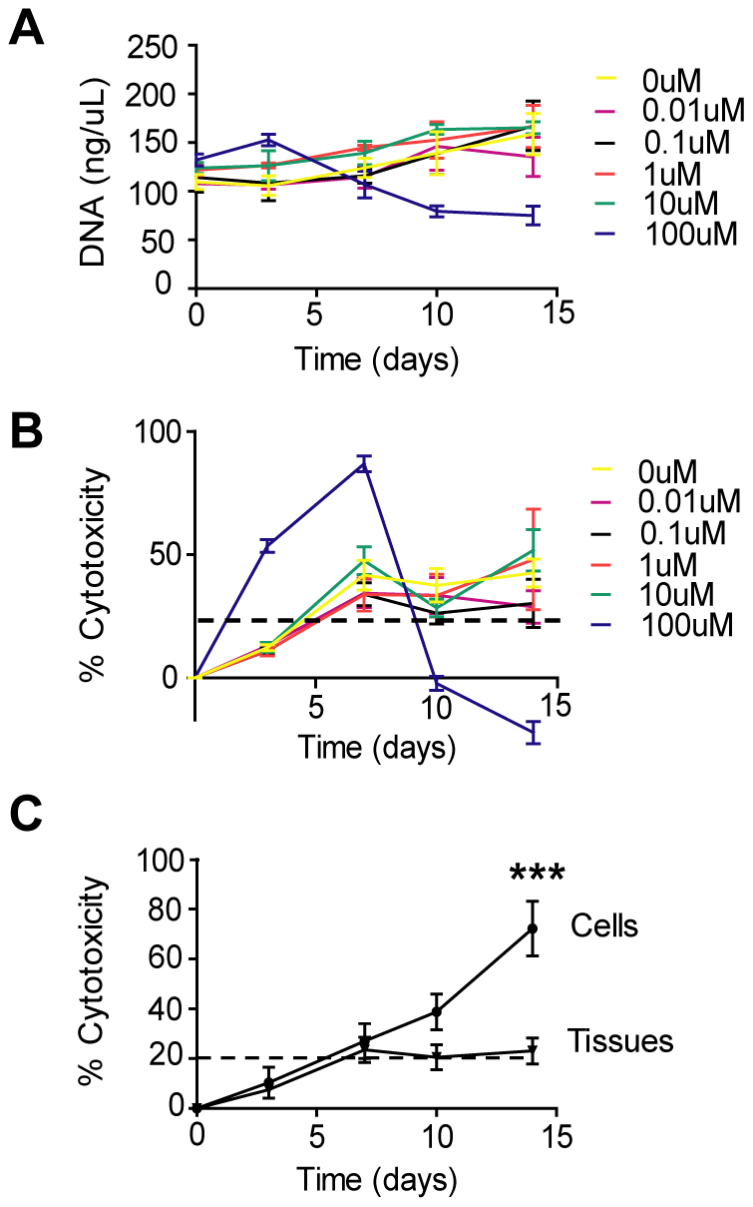
Cytotoxicity assay development for bioengineered 3D tissues. (A) DNA content of bioengineered kidney tissues over time with treatment of a range of cisplatin concentrations. Tissues were grown for 2 weeks and treated with a 5 concentrations of cisplatin from 0.01 µM to 100 µM. A subset of tissues was lysed at 0, 3, 7, 10, and 14 days, for each treatment and DNA content was measured using Picogreen fluorescence. n  =  4. (**B**) LDH secretion over time with treatment of various concentrations of cisplatin. Tissues were grown for 2 weeks and treated with 5 concentrations of cisplatin from 0.01 µM to 100 µM. Supernatant was taken at 0, 3, 7, 10, and 14 days and assayed for LDH activity. n  =  4. (**C**) NKi-2 cell and 3D tissue secretion of LDH over time. NKi-2 cells were seeded at a density of 100,000 cells per well of a 6-well plate and the media supernatant was assayed for LDH secretion 2, 5, 9, 12, and 16 days after seeding. 3D tissues were grown for 2 weeks and the supernatant was assayed for LDH secretion 14, 17, 21, 24, and 28 days after formation of the tissues. ***p<0.001, n  =  4.

Once established, the 3D human kidney tissue system was utilized to evaluate drug-induced toxicity over time. Lactate dehydrogenase (LDH) is an enzyme released by cells upon damage to the plasma membrane and is used often in cytotoxicity assays as it can be measured in the cell culture supernatant over time. However, its usefulness for our 3D cultures was not clear. In order to validate the usefulness of LDH secretion for toxicity measurements with our 3D tissues, we collected supernatant from 3D tissues after treatment with a range of cisplatin concentrations (0 to 100 µM) over a 2 week period and analyzed for LDH activity. Cisplatin is a cancer therapeutic that is highly nephrotoxic with its dose limited by the initiation of renal failure in patients [29]. A subset of 3D tissues at each time point was lysed and DNA content quantified via picogreen fluorescence to establish the relationship between LDH and cell number (**Fig. 4**). The loss of DNA (**Fig. 4A**) followed the same trend as the increase in cytotoxicity measured by LDH secretion (**Fig. 4B**), with the highest concentration of cisplatin causing a significant loss of DNA and a significant increase in cytotoxicity compared to the other concentrations of cisplatin. The LDH value increased at day 3 and 7 indicating changes to the plasma membrane while the [DNA] did not decrease until after those timepoints reflecting the subsequent breakdown of DNA. Taken together this data revealed that assaying LDH release to measure cytotoxicity is relevant to our 3D tissue model.

To further validate the use of LDH for long-term cytotoxicity measurements and to understand the role of background LDH release for future drug studies, we measured the level of secreted LDH in the untreated samples over the same time course we would use for drug testing. Therefore, the 3D tissues were grown for 2 weeks and the 2D NKi-2 cells for 24 hours prior to supernatant sampling at 0, 3, 7, 10, and 14 days, and LDH activity testing. Tissues and cells treated with 1% triton were used as a control for maximal cytotoxic response. The cells and tissues had the same low levels of LDH secretion as measured by % cytotoxicity against control for the early time points (**Fig. 4C**). However, after day 7, the cells in 2D culture overgrew their culture causing cell death that led to a significant increase in the cytotoxicity (greater than 20%) of the cultures by day 14 (**Fig. 4C**). This response did not occur with the 3D tissues as the cytotoxicity remained at approximately 20% and the cell numbers remained relatively steady throughout the 2 week period of culture (**Fig. 4C and 4A**). These data illustrate one of the benefits of the 3D tissue system in comparison to 2D cultures, namely the ability for long-term monitoring of cytotoxicity.

### Drug-Induced Toxicity Testing with Bioengineered 3D Human Renal Tissue Constructs

**Figure 5:**
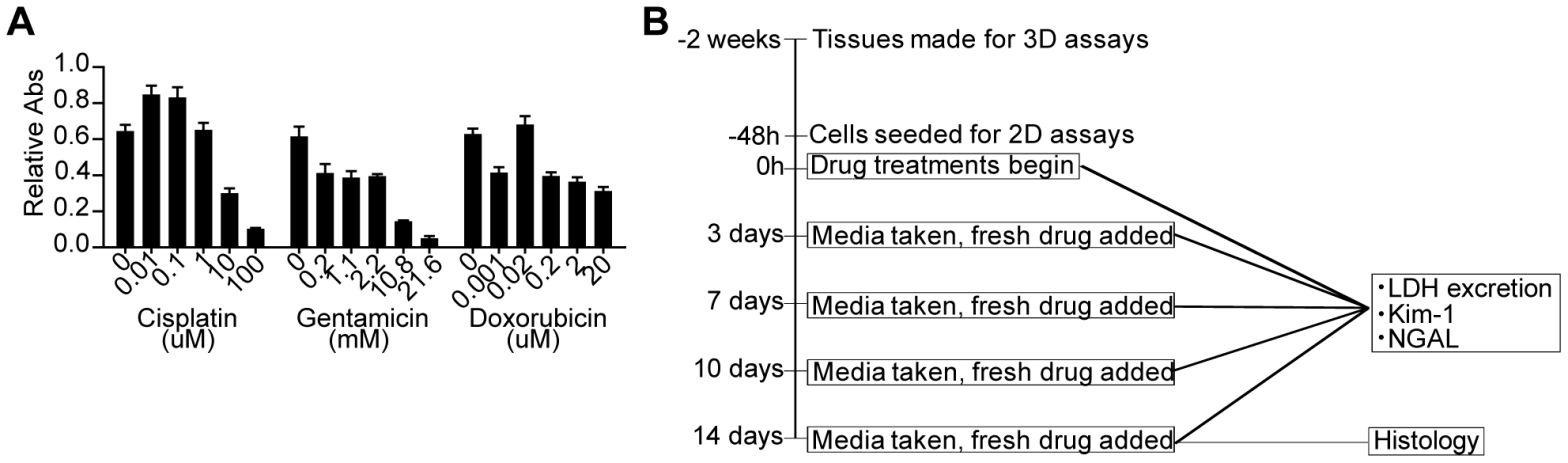
Drug treatment range and strategy. (A) NKi-2 cells were subjected to a range of drug concentrations for 48 hours and cell proliferation was determined by reduction of MTT. n  =  6. (**B**) Schematic of the process of drug treatment followed for both the NKi-2 cells and the 3D tissues.

**Figure 6:**
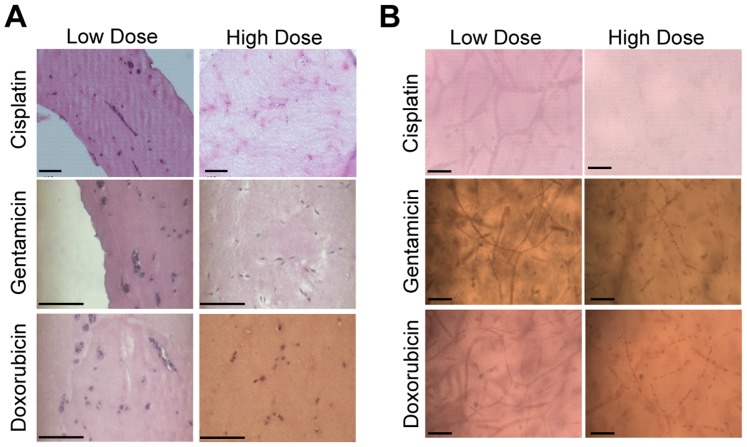
Morphology of 3D tissues after treatment with cisplatin, gentamicin, and doxorubicin. Tissues were fixed after 2 weeks of drug treatment. (**A**) 8 µm sections were stained with hematoxylin and eosin to visualize changes in tissue structure. (**B**) Whole fixed tissues were stained with carmine for whole mount visualization of tissue structure. Low dose refers to the lowest does tested and high dose refers to the highest dose tested. Scale bars  =  50 µm.

**Figure 7:**
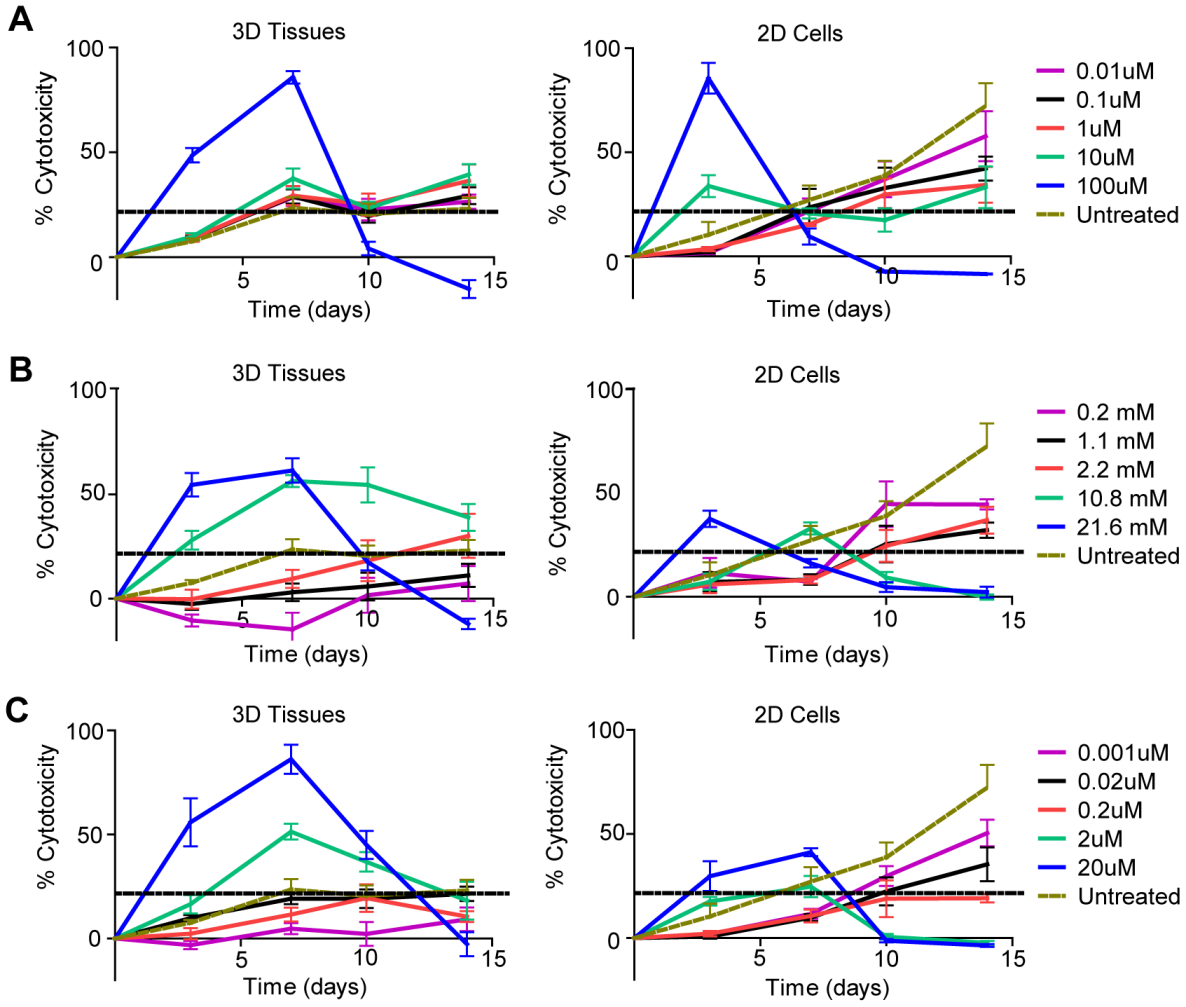
LDH measured cytotoxicity in NKi-2 cells and tissues upon treatment with cisplatin, gentamicin, or doxorubicin. LDH secretion was measured in the supernatant of both NKi-2 2D cell cultures and 3D tissues at 0, 3, 7, 10, and 14 days following treatment with (**A**) 0.01 µM to 100 µM cisplatin, (**B**) 0.2 mM to 21.6 mM gentamicin, or (**C**) 0.001 µM to 20 µM doxorubicin. n  =  7.

In order to validate our bioengineered 3D human kidney tissue model for drug toxicity testing, we first determined drug concentration ranges for cisplatin, gentamicin and doxorubicin capable of causing toxicity for the NKi-2 cells grown in 2D cultures using an MTT assay (**Fig. 5A**). Gentamicin is an aminoglycoside antibiotic also known to be nephrotoxic in patients [30]. Doxorubicin is also a nephrotoxic cancer therapeutic, but its dose limitations are based upon cardiotoxicity [31]. For every drug study, we followed the same timeline; tissues were allowed to grow for 2 weeks and 2D cell cultures for 24 hours prior to the initiation of drug treatment. We then tested the tissue model after 0, 3, 7, 10, and 14 days of treatment with different concentrations of drug and serial sampling of tissue culture supernatant (**Fig. 5B**). Cell culture supernatants were frozen and new culture media with the respective drug dosage was re-applied at each timepoint. Tissues were fixed after the 14 day time point and morphology was examined by both H&E (**Fig. 6A**) and whole mount (**Fig. 6B**). The morphology of the lowest dose treatment for all drugs tested looked similar to the phenotype of untreated tissue (**Fig. 2B**), while the morphology after the highest dose treatments revealed disorganization and loss of structure for both gentamicin and doxorubicin treatments, and complete loss of all cells for the cisplatin treatment. LDH secretion was used to monitor cytotoxicity over time (**Fig. 7**). LDH secretion was different between the 2D cultures of NKi-2 and the 3D tissues. With cisplatin treatment, the 3D tissues had greater than 20% cytotoxicity at a concentration ≥ 1 µM and that peaked for 100 µM on day 7 while in 2D, the cytotoxicity only exceeded the untreated controls at ≥ 10 µM and peaked for 100 µM on day 3 (**Fig. 7A**). In animal studies, cisplatin has been shown to exert toxic effects from 1 to 3 days after treatment depending upon dosage [32], [33], [34]. For gentamicin, the 3D tissues had greater than 20% cytotoxicity by day 7 at ≥ 5 mg/mL and the initiation of chronic toxicity at day 14 for 1 mg/mL while in 2D, only the 10 mg/mL concentration exceeded the untreated controls at day 3 (**Fig. 7B**). Finally, for doxorubicin, the 3D tissues had greater than 20% cytotoxicity at concentrations ≥ 2 µM that peaked on day 7 while in 2D, only the 20 µM concentration exceeded the untreated control by day 7 (**Fig. 7C**). These differences could be due to variations in the uptake and/or, in the case of cisplatin, the metabolism of the drug between 2D and 3D cultures.

The difference in cytotoxicity effects between 3D tissues and 2D cell cultures became more obvious when the drug concentration responsible for 50% cytotoxicity (LD_50_) was compared between 2D cells and 3D tissues for all three drugs (**Table 1**). The LD_50_ value was calculated using the LDH % cytotoxicity values from at least 7 different samples at the time of peak toxicity. While the cisplatin LD_50_ value was similar between 2D and 3D, potentially reflecting the severe toxicity of cisplatin for renal proximal tubule cells, the LD_50_ value for both gentamicin and doxorubicin was significantly different between 2D and 3D, with the 3D tissues being much more sensitive than the cells in 2D culture. Taken together, these data indicate that our 3D tissue model is useful for the detection of nephrotoxicity, is as or more sensitive than 2D cell culture, and is capable of monitored cell responses over extended time frames unlike 2D cell culture.

**Table 1:** LD_50_ values for 3D tissues and 2D cell cultures treated with cisplatin, gentamicin, or doxorubicin.

Model System	Cisplatin	Gentamicin	Doxorubicin
hRPTEC	39.6 µM [39]		11.2 µM [39]
HK-2	>1 mM [12]	>1 mM [12]	1 µM [12]
NKi-2 Cells (LD_50_)	17 µM (n = 7)	22 mM (n = 7)	>20 µM (n = 7)
NKi-2 Tissues (LD_50_)	25 µM (n = 19)	9 mM (n = 7)	2 µM (n = 7)

### Biomarker Measurement of Drug-Induced Toxicity in Bioengineered 3D Human Renal Tissue Constructs Compared to 2D Cell Culture

**Figure 8:**
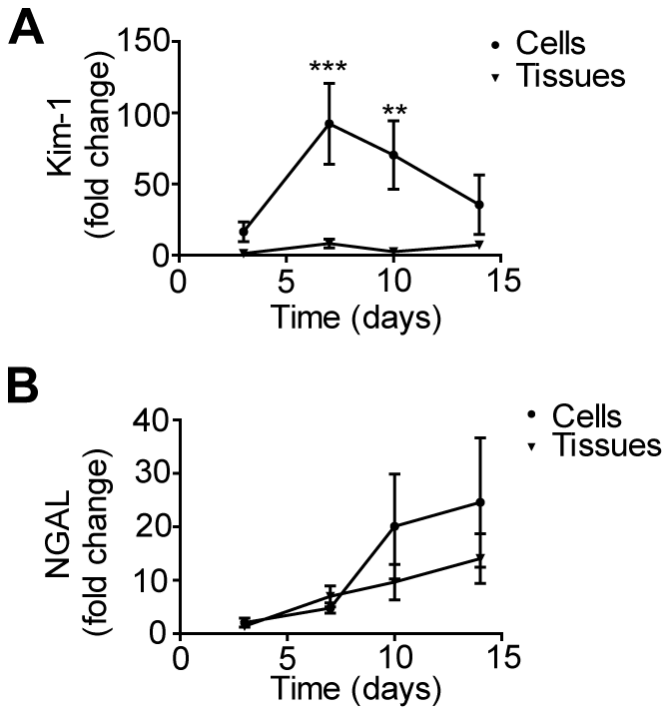
Kim-1 and NGAL secretion in untreated NKi-2 cells and 3D tissues. Untreated NKi-2 cells were grown for 24 hours followed by media sampling at 0, 3, 7, 10, and 14 days. The media was assayed for the presence of (**A**) Kim-1 or (**B**) NGAL by ELISA. NKi-2 3D tissues were grown for 2 weeks followed by media sampling at 0, 3, 7, 10, and 14 days and assayed for the presence of (**A**) Kim-1 or (**B**) NGAL by ELISA. **p<0.01, ***p<0.001, n  =  7.

**Figure 9:**
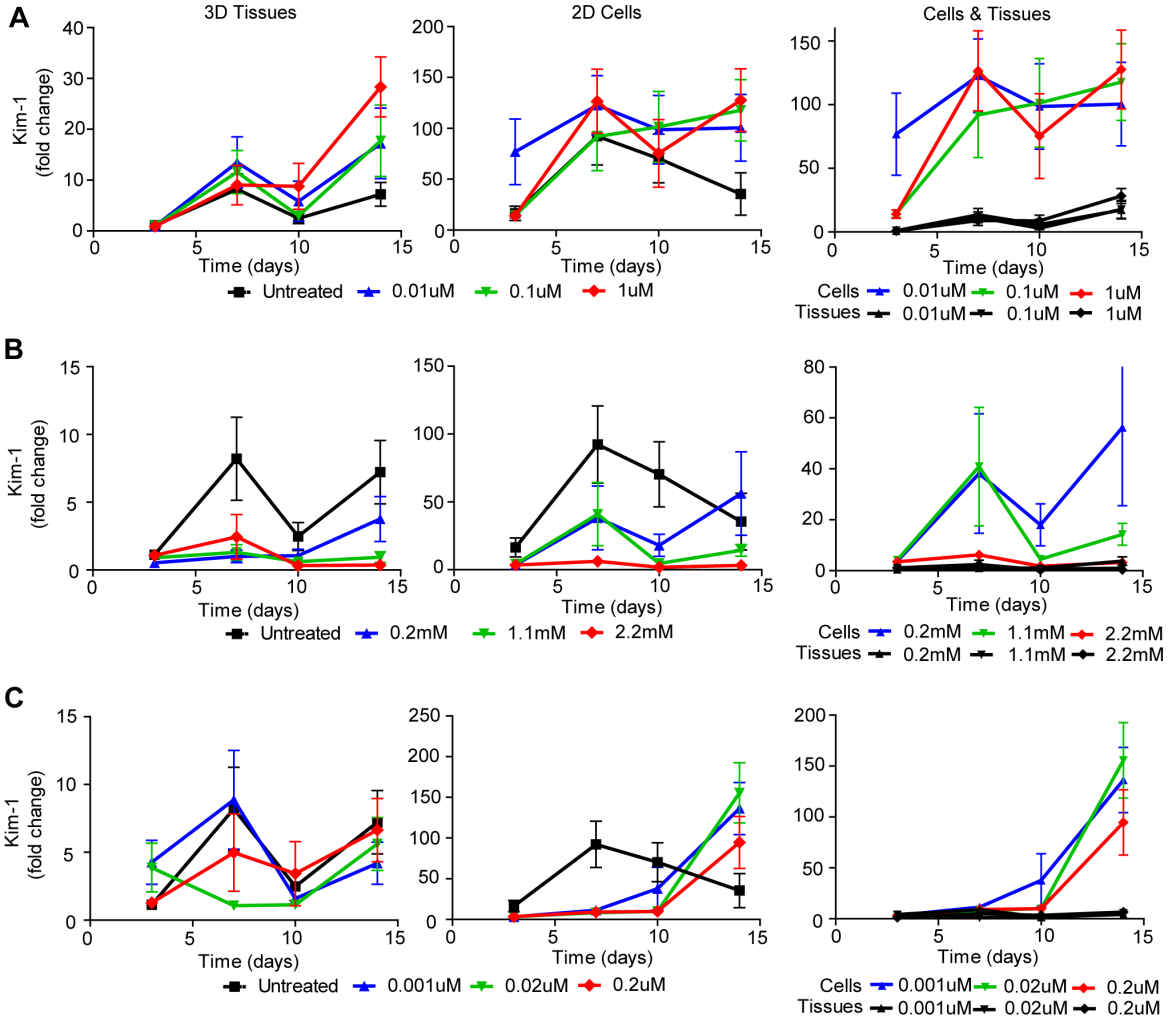
Kim-1 measured cytotoxicity in NKi-2 cells and tissues upon treatment with cisplatin, gentamicin, or doxorubicin. Kim-1 secretion was measured by ELISA in the supernatant of both NKi-2 2D cell cultures and 3D tissues at 0, 3, 7, 10, and 14 days following treatment with (**A**) 0.01 µM to 1 µM cisplatin, (**B**) 0.2 mM to 2.2 mM gentamicin, or (**C**) 0.001 µM to 0.2 µM doxorubicin. n  =  7.

**Figure 10:**
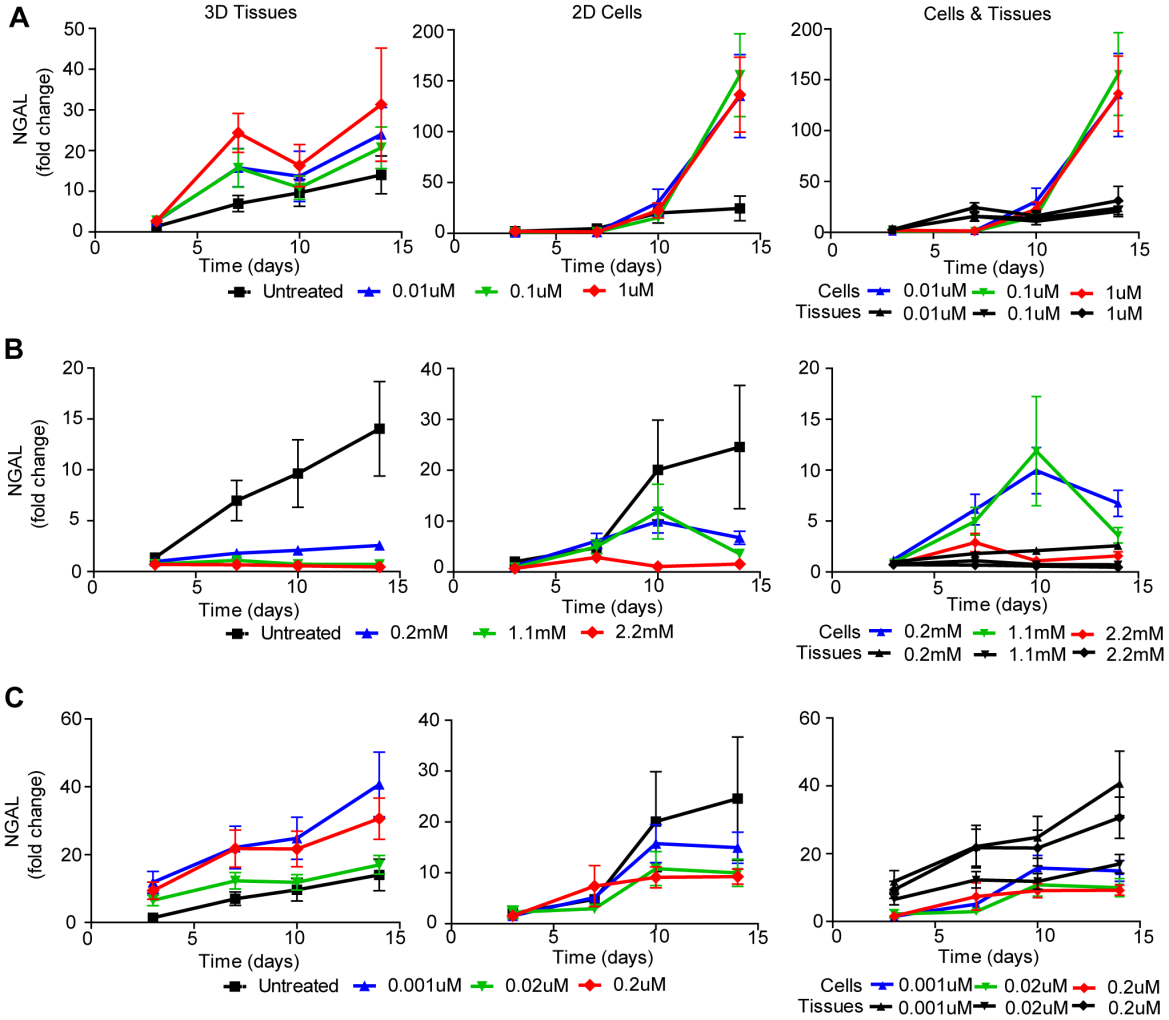
NGAL measured cytotoxicity in NKi-2 cells and tissues upon treatment with cisplatin, gentamicin, or doxorubicin. NGAL secretion was measured by ELISA in the supernatant of both NKi-2 2D cell cultures and 3D tissues at 0, 3, 7, 10, and 14 days following treatment with (**A**) 0.01 µM to 1 µM cisplatin, (**B**) 0.2 mM to 2.2 mM gentamicin, or (**C**) 0.001 µM to 0.2 µM doxorubicin. n  =  7.

Lastly, we examined whether we could exploit the use of pre-clinical biomarkers [35], [3] to monitor cytotoxicity in our 3D human renal tissue constructs. Two such biomarkers are kidney injury molecule-1 (Kim-1) and neutrophil gelatinase-associated lipocalin (NGAL). Kim-1 is a transmembrane protein that is not found in healthy human kidney tissue but is strongly upregulated in proximal tubule epithelial cells upon injury [36]. NGAL is a cytosolic protein released early upon proximal tubule cell injury [37]. ELISAs were used to assess the secretion of both biomarkers into the cell culture supernatant of untreated NKi-2 cells and untreated 3D tissues to establish baseline expression (**Fig. 8**). The 3D tissues and NKi-2 cells were treated the same as for the untreated LDH experiments (**Fig. 4C**). The fold change in expression of Kim-1 (**Fig. 8A**) increased significantly over time in the 2D cell cultures while remaining steady in the 3D tissues. The increase in 2D cell culture correlated with the increase in cytotoxicity seen by LDH in untreated cultures (**Fig. 4C**).This may reflect the cell death that occurs over time in 2D culture due to increasing cell confluency and indicate that Kim-1 may be a good marker for 3D tissues but not for 2D cell culture due to the high background expression level. NGAL expression did not change as significantly as Kim-1 over time in untreated samples and there was no significant difference between the NKi-2 cells in culture and the 3D tissues over time (**Fig. 8B**). Kim-1 (**Fig. 9**) and NGAL (**Fig. 10**) levels in the cell culture supernatant were then assessed after exposure of the cultures to cisplatin, gentamicin, and doxorubicin. Expression of Kim-1 at the two highest treatment levels dropped immediately to zero for all three drugs, reflecting the highly toxic nature of the highest drug concentrations and are therefore not shown on the graphs. For gentamicin 2D cultures and 3D tissues, Kim-1 levels were below untreated (control) levels for all drug concentrations (**Fig. 9B**). Thus, for gentamicin, Kim-1 was not a good bio-marker of drug-induced toxicity. However, for cisplatin treated cultures, Kim-1 expression in the supernatant increased above untreated controls after exposure to lower concentrations at later time points for both 2D culture and 3D tissues (**Fig. 9A**). This was also seen with doxorubicin treatment of 2D cultures (**Fig. 9C**). This may indicate a cumulative tissue injury with repeated dosing at these low drug concentrations; a situation more relevant to clinical practice than acute cytotoxic effects at high doses. Importantly, for all three drugs, 2D cell culture secretions were higher than their 3D tissue counterparts. This may be due to the ease of release in 2D compared to 3D where Kim-1 may get retained in the ECM components.

NGAL secretion followed a similar pattern as Kim-1 over time as a good indicator of cisplatin toxicity (**Fig. 10A**) but not gentamicin toxicity (**Fig. 10B**). However, NGAL was also a good indicator of toxicity for doxorubicin in 3D tissues, unlike Kim-1 (**Fig. 10C**). Unlike Kim-1, there was not as big a difference between 2D cultures and 3D tissues in terms of overall NGAL secretion. In fact, doxorubicin 3D tissues had a greater increase in NGAL secretion over time than did the same treatment groups in 2D. Sasaki et al has shown that rats dosed with cisplatin or gentamicin can take up to 7 days to have increased expression of Kim-1 and NGAL in their urine at high doses [38], indicating that our time course may be relevant to *in vivo* situations. Our data also shows the usefulness of our 3D model over 2D culture for the interpretation of biomarker data as the 3D tissues do not have as high a level of background expression as the 2D cultures.

## Discussion

The incredibly poor success rate of drugs that enter clinical trials, a better understanding of the limitations of 2D cell culture, and initiatives to reduce the number of animals used for drug testing, have driven a recent push in the development of 3D human tissue models for the study of drug toxicity. It is hypothesized that these 3D human tissue models will better recapitulate the human *in vivo* response to drugs. Here we have characterized a new and unique bioengineered 3D model of human kidney tissue and applied it to the testing of drug-induced nephrotoxicity in comparison to 2D cell culture. We have shown that the 3D tissue model has a different timing of toxicity induction compared to 2D culture and is more sensitive to drug dosage, having lower LD_50_ values. Furthermore, we have shown the utility of this model for chronic toxicity testing and examined the relevance of pre-clinical markers for monitoring toxicity in 3D.

Previous 2D models of nephrotoxicity have relied upon primary proximal tubule cells [5], [39]. Presumably this allows for the monitoring of toxicity in a cell in a state very close to the *in vivo* state in terms of gene expression. However, primary cells are not a constantly renewable source, requiring re-isolation from other patients due to their inability to proliferate over numerous passages [40] and the changes in gene expression they undergo relatively quickly upon *in vitro* growth [41]. To overcome this problem, our bioengineered 3D human renal tissue described here consists of hTERT immortalized human renal epithelial cells of primarily proximal tubule origin. It has been shown that immortalized cells lose the expression of organic anion transporters and other proteins compared to primary cells [42], [22], potentially impairing their suitability for toxicology testing. However, the use of immortalized cells for toxicology would provide genetic consistency compared to the variability inherent in using primary cells from different patients and may allow for the production of a renewable, stable collection of cells for testing different disease states. hTERT immortalization of human renal epithelial cells has been shown to not have a significant effect upon their function [43] and we have shown in our studies that our hTERT immortalized renal epithelial cells have more *in vivo* like renal function in 3D culture compared to 2D culture, maintain the expression of OAT1 and OAT4, and renew the transcription of megalin when grown as a 3D tissue. An additional problem with the use of immortalized cells lines is their constant proliferation which would make them unsuitable for long term chronic studies. This is evident in our data in which LDH increases over time in 2D without drug treatment. However, this problem was not evident in 3D tissue culture. They did not over proliferate, destroying the bioengineered tissue, even after 8 weeks of culture. This may be the result of signaling events between the ECM and the cells or a result of their formation into 3D structures. Further examination of the 3D model may reveal the reason for the apparent senescence of the cells in 3D compared to 2D and further research into drug toxicity mechanisms should provide further evidence of the utility of immortalized kidney epithelial cells for *in vitro* drug testing.

One of the best ways to gauge the toxic effect of a compound on a system and compare it to other systems is with an LD50 value. Significantly, toxicity data in our 3D model revealed a decrease in the LD50 for both gentamicin and doxorubicin compared to both our NKi-2 cells in 2D and previously published values for hRPTEC [39]. Additionally, while cisplatin LD50 was similar between 2D and 3D, with the 3D being higher, both values were lower than published values for hRPTEC [39] and HK-2 cells [12]. While it is difficult to translate this into *in vivo* relevance, previous studies of gentamicin toxicity in 2D have shown that extremely high doses and prolonged periods of exposure are necessary to achieve maximum toxicity compared to what is required in patients [44], [12]. Taken together, this indicates that our bioengineered 3D model is more sensitive than the cells used in it in 2D and hRPTECs. In the future, this data may be used to better direct the dosing studies performed in animals thereby reducing the number of animals used in a study. Furthermore, the inclusion of 3D model data with animal data may be able to better predict the toxicity of a compound and the acceptable dose range in patients.

Unlike *in vivo* kidney injury testing that can examine BUN and serum creatinine levels, *in vitro* testing is limited to either non-specific markers of cell death such as LDH or, in the case of the kidney, specific markers of renal epithelial cell injury such as Kim-1 and NGAL. To better reveal the usefulness of our bioengineered 3D model for pre-clinical drug testing, we looked at the secretion of these pre-clinical biomarkers. The use of these biomarkers *in vitro* may be limited as NGAL has been shown to be upregulated upon proximal tubule cell preparation [45] and Kim-1 may not be properly expressed in cells in 2D culture [46]. Our cells in 2D culture expressed significantly higher levels of Kim-1 than in 3D tissues. When this is combined with our data revealing more *in vivo* like function in 3D, it suggests that the cells may behave more like their *in vivo* counterparts when allowed to form 3D structure. Additionally, the loss of viable cells within the model system due to high toxicity was evident by the loss of Kim-1 expression. The ability to use pre-clinical biomarkers with the 3D human renal tissue constructs indicates both their similarity to *in vivo* tissue and their usefulness for future nephrotoxicity studies.

We have shown that a 3D model of human renal tissue is feasible and can be applied to pre-clinical drug testing with approved biomarkers. While the use of 3D models for nephrotoxicity studies has been explored using mouse tubules imbedded in hydrogels [47], [48], this is the first model to not only look at function of human kidney epithelial cells in a hydrogel system, but to also apply it to the determination of nephrotoxicity. This model system has the potential to limit the amount of animals needed in pre-clinical testing and may better inform upon dosing in patients. Further studies examining the expression of transporters and drug metabolizing enzymes and the mechanism of drug action in the cells in the 3D model will serve to better validate the system and reveal its suitability for studying drug toxicity mechanisms.

## Acknowledgments

We are grateful to Dr. Jing Zhou and Dr. Wassim Al-joudi for the immortalized human renal cortical epithelial cells used in this study. We are also thankful to Dr. Balajikarthick Subramanian, Wei-che Ko, Aiai Ran, Erika Parisi, and Greg McDonough for their technical help and support.
