# Correction: Bioengineered 3D Human Kidney Tissue, a Platform for the Determination of Nephrotoxicity

**DOI:** 10.1371/annotation/fb32f1b8-7397-40be-bbf9-b80e67763043

**Published:** 2013-10-24

**Authors:** Teresa M. DesRochers, Laura Suter, Adrian Roth, David L. Kaplan

Errors were introduced during the preparation of this article for publication. This article has been republished to replace incorrect figures. 

